# Effects of Covid-19 confinement on the mental health of children and adolescents in Spain

**DOI:** 10.1038/s41598-021-91299-9

**Published:** 2021-06-03

**Authors:** Juan Pablo Pizarro-Ruiz, Nuria Ordóñez-Camblor

**Affiliations:** 1 Department of Educational Sciences, University of Burgos, 09001 Burgos, Spain; 2 Department of Health Sciences, University of Burgos, 09001 Burgos, Spain

**Keywords:** Human behaviour, Comorbidities

## Abstract

In Spain, in order to control COVID-19 transmission, one of the strictest confinement measures in the world for children and teenagers has been implemented. From 14 March to 26 April 2020 underage Spaniards were not allowed to leave their homes, except for reasons of force majeure. This could have consequences on their mental health in both the short and the long term. Thus, the aim of the present study was to explore the consequences of confinement on the mental health of Spanish children and teenagers, at the time when minors had been locked down in their homes between 8 and 10 days. The sample was composed of 590 confined Spanish children and teenagers between 8 and 18 years old. The scales of Depression, Self-esteem, Anxiety, Problems with Emotional Regulation, Rage Control Problems, Integration and Social Competence, Somatic Complaints, Rebellious Behaviour, as well as Awareness of the Problems of the Assessment System for Children and Adolescents (SENA) were used. The results revealed that, during confinement, children and adolescents showed emotional and behavioural alterations. This study, as far as we know, is the first one to explore the psychological consequences of lockdown in minors while it was taking place, with them being the ones directly assessed.

## Introduction

In order to control the contagion of the COVID-19 disease, the Spanish Government implemented strict domestic quarantine policies. On 14 March 2020 the State of Alarm and confinement for the entire population, including children, was decreed. When more than 7500 people had tested positive for COVID-19, 293 were admitted to the ICU and 136 people died^1^.

In relation to confinement measures, Spain has been one of the most restrictive countries, concerning permission for minors to leave their houses. From 14 March to 26 April 2020 minors in Spain were not allowed to do this. This meant they were in lockdown at home for six continuous weeks, when the adverse psychological effects on children and teenagers are yet to be determined. In this regard, the data in China indicate that the consequences on the levels of depression during quarantine were greater in teenagers than in adults^2,3^.

It is hoped that even after such disasters, most people are resilient and do not develop psychopathological problems. However, some groups can be more vulnerable to the psychosocial effects of pandemics^4^. In children at key stages, the interruption of social and educational activities for many months can have a higher impact on their development. Recent investigations on the consequences of stressful situations in the mental health of small children show that anxiety, depression, lethargy, damaged social interaction and poor appetite are the most usual psychological manifestations, while at the physiological level a weakened immune system can be observed^5–8^.

Recently, Fegert et al.^9^ established the impact of the phases of the pandemic on the mental health of children and adolescents. They include in their study certain risk factors and difficulties that children have to face in the first phase of the COVID-19 pandemic: children have not been allowed to use the usual playgrounds, social group activities are forbidden, sports clubs are closed, social relationships have been strongly limited to close relatives, contact with peers has been forbidden, school closures, loss of education time, and loss of daily structure. In addition, their families have also faced multiple challenges: reorganization of daily life, coping with the stress of quarantine and social distancing, homeschooling, increased pressure to work from home and care for school-age children at home at the same time, reduced freedom and privacy, and economic concerns due to business closures. In addition, in Spain, childcare support—such as grandparents—disappeared unless they were cohabitants. This can result in enormous stress and psychological distress for all family members; and increase family and marital conflict. All this represents a dangerous accumulation of risk factors for mental health problems in children and adolescents of enormous proportions: reorganization of family life, massive stress, fear of death of family members, economic problems, loss of support systems, lack of opportunities for leisure and escape, limited access to health services, lack of socialization, lack of access to schools and sports activities. In this line, also in phase 1, Wang et al.^2^ made their evaluation and found that the impact of the outbreak on the mental health of the children was moderate to severe in 54% of the participants, with depressive symptoms and anxiety being the most frequent.

Regarding the emotional consequences of events linked to health, Sprang and Sigman^10^, analysed the effects of pandemics on 398 families, discovering that children who were isolated or in quarantine during pandemic illnesses were more likely to develop acute stress disorder, adjustment disorder and mourning. Thirty percent of the children that were isolated or in quarantine met the clinical criteria for post-traumatic stress disorder and attended mental health services. Recently, a preliminary study conducted in the province of Shaanxi during the second week of February 2020^11^, in which the Chinese population were confined, showed that the most common psychological and behavioural disorders of 320 children and teenagers between 3 and 18 years old were: bonding problems, distraction, irritability and being afraid to ask questions about the epidemic. Regarding differences according to age, the youngest, between 3 and 6 years old, were more likely to develop bonding problems and fear that the members of the family could contract the illness, while children between 6 and 18 years old showed more attention problems and persistent inquiry^11^.

Also in China, Zhou et al.^3^ conducted an investigation on teenagers between 12 and 18 years old, discovering a high prevalence of anxiety (43.7%) and depression (37.4%) during the COVID-19 outbreak, especially in females. Along the same line, Pisano et al.^12^ conducted a study in Italy in which they evaluated, through the parents, the impact of the confinement in minors between 4 and 10 years old, in the form of a questionnaire created ad-hoc. The results showed regressive behaviour, such as the wish to sleep with their parents; enuresis episodes; a worsening in their vocabulary; and fears that were inexistent before. In regard to emotional or behavioural changes, 53.5% of the children showed more irritability: 21.2% showed continuous mood changes; 20% revealed trouble sleeping; and 34.3% were more nervous.

Even though, as shown, it is expected that a few of the children and teenagers react in a resilient way to this crisis, the risk is real and needs attention^13^. Minors, in addition to the aforementioned, can experience fear of the virus, frustration, boredom, lack of socialization with friends and teachers and lack of room at home, leading to problematic consequences that can go beyond the quarantine period^14^. In Spain, children and adolescents stopped attending school from the beginning of the confinement (March 14th) to (predictably) the beginning of the next school year in September 2020. If also, we add to this that the mental health services for minors must face the new challenges that the COVID epidemic has brought, and without guidelines adapted to the new circumstances or that are restricted to the worst cases, addressing these consequences is even more uncertain^14,15^.

Furthermore, empirical studies in this area are very scarce. A review by Nearchou et al.^16^ found only 12 investigations on child and adolescent mental health during the COVID-19 pandemic analyzing eight databases (PsycINFO, MEDLINE, CINAHL, Scopus, PubMed, EMBASE, ERIC and the WHO database on COVID-19). All were quantitative cross-sectional designs. Two of these 12 studies used parents as informants^11,17^, four also used adults in their samples and the sample of minors did not exceed n = 34^18–21^ and only two studies—in China—evaluated children^22,23^ and four—in the USA, Turkey and China—adolescents^3,24–26^. It seems clear that more research is needed in this area.

In recent months, a number of studies have appeared that reflect the growing interest in the mental health of children and adolescents due to COVID-19. Worthy of note are studies such as that of Breaux et al.^27^ who followed 338 adolescents by assessing them before COVID-19 and during the government's stay-at-home recommendations in the USA. They found that adolescents experienced an increase in depression, anxiety, slow cognitive tempo, inattention and oppositional defiant symptoms^27^. Or in Europe, Ravens-Sieberer et al.^28^ evaluated 1586 families of German children and adolescents, finding that minors presented more mental health and quality of life problems after the COVID-19 pandemic. However, in Germany the incidence and mortality rates were low compared to countries such as Spain, Italy or China, and their lockdown measures were less severe than in these countries^28^ (e.g., in Spain children were forbidden to leave the house and in Germany they could). Also, when the authors compare the impacts cross-culturally, children and adolescents in Germany do not seem to be affected as negatively as in other countries. Despite this, German children feel significantly burdened by confinement, social distancing and home schooling measures. Also in Europe, Francisco et al.^29^ evaluate the initial impact of quarantine on parents in three countries (Spain, Portugal and Italy). They found, by means of an ad-hoc questionnaire, that parents found their children to be more irritable, lonely, restless and uncomfortable.

Despite research findings indicating the negative effect of confinement, research on its impact on children and adolescents remains very scarce^29–31^. Despite investigation findings indicating the negative effect of confinement, research on its impact on children and adolescents remains very scarce. Even more so in countries where confinement has been severe and forced to prevent transmission of COVID-19.

In this context, the main goal of the present report is to analyse the consequences of strict confinement on the mental health of children and teenagers, at the time when minors had been confined to their homes between 8 and 10 days, being the ones directly evaluated, and while it was taking place. All of this has occurred in one of the countries with the most restrictive confinement measures and which has met all the restrictions of movement in a stricter way, according to the mobility data of Google and Apple^32–34^. This research is, as far as we know, the one that performs the earliest assessment of the confinement of minors by COVID-19. We know that early impacts and reactions to catastrophes are important in predicting the onset of future psychological disorders. For example, a study conducted among Norwegian child survivors of the tsunami in 2004 reported that the immediate subjective response to it, was correlated to Posttraumatic Stress Disorder reaction 6–8 months later^35^.

Furthermore, a bigger number of variables linked to the mental health of minors is depicted, in comparison to the reports on anxiety and depression that have been published in recent studies of children and teenagers conducted in China^3,36^. In this way, first prevalence rates are gathered and compared to data from minors in quarantine with the scales of the technical manual of the SENA^37^ (Assessment System for Children and Adolescents), which is the resource used in the present study to evaluate the state of mental health of minors.

The second goal is to investigate if confinement has affected the levels of anxiety, depression, self-esteem, rebellious behavior, somatic complaints integration and social competence, rage control problems, as well as greater emotional regulation problems in children or teenagers. Liu et al.^22^ found different impacts of the pandemic on adolescents than on children, with adolescents reporting worse mental health. Something similar is found by Zhou et al.^3^, although they compare college adolescents with elementary school children. However, Ravens-Sieberer et al.^28^ find that younger children are more affected by the pandemic than adolescents.

Lastly, the third goal is to differentiate between the mental health of children and adolescents based on gender and level of education, since these were some of the main risk factors for anxiety and depression in Chinese adolescents^3^. Regarding gender differences, the results are controversial. Some studies find that female children and adolescents report worse mental health during confinement^3,28^. But others find no gender differences on the mental health of confined minors^22,23^.

Our main hypotheses were:

Hypotheses 1: The sample of confined children and adolescents presents worse mental health than those who were not confined.

Hypotheses 2: Confined adolescents present worse mental health than confined children.

Hypotheses 3: Female children and adolescents present worse mental health than male children and adolescents.

Hypotheses 4: Children and adolescents in courses with highter academic pressure present worse mental health than children and adolescents with less pressure.

## Methods

### Participants

The sample consisted of 788 minors, 440 of whom were between 8 and 12 years old and 348 between 13 and 18 years old. After removing the participants that had not answered 100% of the raised items, 590 minors were left, 325 between 8 and 12 years old and 265 between 13 and 18 years old (teenagers). It is an incidental sample, instead of a random one. The selection of participants was carried out through an anonymous survey conducted on the Internet, social networks and the press.

In the case of the children (between 8 and 12 years old) 158 were girls (51.7%) and the average age was 9.95 years old (*SD* = 1.40). Regarding the place of residence, they were from all the Spanish communities; the most represented was Castilla y León, where more than half of the participants lived (71.75%), followed by The Basque Country (7.7%) and Madrid (6.5%). A total of 81.5% lived in urban areas and 43.4% were studying the intermediate level of primary education. 25.8% had no siblings, 54.5% had one sibling and 19.7% had two or more siblings. At the time of confinement, 17 children (5.2%) lived with only one person in their home, 82 with two people (25.2%), 166 with three people (51.1%) and 60 with more than three (18.5%). 20.9% had a garden in their home, 39.1% had a terrace or balcony and 39.7% had neither. The average square meters of their home was 113.2 (SD = 71.1). 17.8% had a dog in their home.

In the sample of the teenagers (between 13 and 18 years old), 168 were female (63.4%), with an average age of 15.42 years old (*SD* = 1.70). They also came from all the Spanish communities, being Castilla y León the most represented again (55.8%), followed by Andalucía (10.9%) and Madrid (6%). 66.0% lived in urban areas, 57.7% studied compulsory secondary education, 33.6% high school and 8.7% vocational training. 19.6% had no siblings, 58.1% had one sibling and 23.3% had two or more siblings. At the time of confinement, 19 adolescents (7.2%) lived with only one person in their home, 59 with two people (22.3%), 142 with three people (53.6%) and 45 with more than three (17.0%). 23.4% had a garden in their home, 42.3% had a terrace or balcony and 34.3% had neither. The average square meters of their home was 119.0 (SD = 67.0). 22.3% had a dog in the home.

The inclusion criteria were:

1. To be between 8 and 18 years of age
2. Parents or legal guardians consent to their children's participation

The exclusion criteria were:

1. Not to be between 8 and 18 years of age
2. Parents or legal guardians do not consent to their children's participation.

### Instruments

Assessment System for Children and Adolescents^37,38^ (SENA) is a resource focused on the detection of a wide range of emotional and behavioural disorders from 3 to 18 years old. It allows one to detect areas of vulnerability that predispose people to develop psychopathological problems and the presence of psychological resources that can act as protective factors to different problems. In our study, self-reporting questionnaires aimed at Elementary (6–12 years old) and High School (13–18 years old) students were used, and some of them were chosen so that they did not take too much time, keeping in mind the state in which the minors were. We selected nine dimensions from different areas of mental health: (a) Internalized problems: Depression (“I'm sad”), Anxiety (“I'm nervous”), and Somatic Complaints (“I feel sick”); (b) Externalized problems: Rebellious Behaviour (“I do what I want to do even if I am punished”) and Rage Control Problems (“I scream when I get angry”); (c)Vulnerability areas: Problems with Emotional Regulation (“My mood changes a lot during the day.”); (d) Psychological resources or protective factors: Self-esteem (“I like how I am”), Awareness of Problems (“I'm having a hard time and I need help”), and Integration and Social Competence (“When I have problems there are people who listen to me”). It shows a response format of a 5-point Likert-type scale. Both children and teenager scales were reliable, producing *α* Cronbach internal consistency rates between 0.754 and 0.914. They present scores in their technical manual on both normal population and clinical samples (anxiety, depression, learning difficulties, *ADHD*, etc.).

SENA has shown suitable psychometric traits referring to evidence of validity, as well as test–retest reliability and internal consistency on both normal and clinical population^38^. In addition, the SENA has been related as expected to a wide variety of assessment instruments such as the BASC-2 (Behavior Assessment System for Children^39^), BRIEF-P (Behavior Rating Inventory of Executive Function-Preschool Version^40^), BRIEF (Behavior Rating Inventory of Executive Function^41^), PAI-A (Personality Assessment Inventory-Adolescent^42^), TAMAI (Multifactorial Adaptive Test of Childhood Adaptation^43^), CECAD (Educational-Clinical Questionnaire: Anxiety and Depression^44^) and STAXI-CA (State-Trait Anger Expression Inventory for children and adolescent^45^) demonstrating adequate validity, understanding, and content of the items^38^.

None of the variables show relevant influence of gender, age, socioeconomic status of the family or the educational level of the parents^37^. Despite the fact that the evaluation tool is validated from 3 year olds on up, in the present work the sample for 8 year olds on up has been chosen, so that the children were able to adequately answer the questions asked them on their own, without adult collaboration^38^. In addition, for younger children (7 year olds or younger), the SENA assessment is conducted in an interview format, not self-report^37^.

### Procedure

A cross-sectional study was conducted through surveys between 22 and 25 of March 2020 (after 8–10 days of confinement at home) by means of an anonymous survey carried out on the Internet, social networks and press, using the onlineencuesta platform (https://www.onlineencuesta.com/), which took approximately 15 min. To try to avoid sample bias, we sent the evaluation questionnaires to multiple destinations and through very different platforms, in order to impact as many people as possible and with as much variability as possible. At first, we sent it, for example, to national news agencies, national and regional newspapers that published the links on their websites, to more than 20 "educational influencers" (primary or secondary school teachers with websites or Youtube channels about education with a large number of followers) some of whom disseminated it in their media and social networks, to more than 500 students from four different Spanish universities, or to five sports clubs in three different cities. In all of them, we urged people not only to have their children participate, but also to disseminate it to as many people as possible.

The investigation was sanctioned by the Research Ethics Committee of the University of Burgos. All parents or legal guardians gave voluntary informed consent to the participation in the study, which was explained to them as an investigation of the effects of confinement on the physical and emotional condition of minors. The study complied with the ethical criteria on research with human beings (informed consent and the right to information, personal data protection and guarantees of privacy, non-discrimination, gratuity and the possibility of leaving the study at any stage).

### Data analysis

First, we analyzed the percentage of children and adolescents scoring moderately high (between one and two standard deviations above the mean) and high (more than two standard deviations).

Then, we conducted a one-sample Student's t test to compare the confined children and adolescents with the SENA validation sample (Hypothesis 1). Effect sizes were estimated using *Cohen's* *d* (low effect size: d = 0.2, medium: d = 0.5, high: d = 0.8)^46^.

Next, a MONAVA was carried out to compare the influence of confinement on the mental health of children vs. adolescents (Hypothesis 2).

Finally, individual ANOVAs were conducted for each of the mental health dimensions assessed to establish differences between children and adolescents, as a function of gender and school year (Hypotheses 3 and 4). For the school year, the post-hoc tests were DMS tests. Effect sizes were estimated using *Partial* *eta* *squared* (low effect size: *η*_*p*_^2^ = 0.01, medium: *η*_*p*_^2^ = 0.059, high: *η*_*p*_^2^ = 0.14)^46^.

## Results

### Prevalence

The correction criteria of the technical manual of SENA^37^ are used in the study of prevalence. Table 1 presents the number and percentage of Spanish children and teenagers in strict confinement who are between 1 and 2 standard deviations above average, more than 2 standard deviations above average, and the ones that scored higher than the clinical sample of SENA scales. In the aspects of self-esteem, integration and social competence and awareness of the problems, the distance in standard deviations is below average, and those participants who score lower than the SENA clinical sample are also shown.

**Table 1:** Prevalence in mental health problems in children and teenagers in Spain during confinement.

SENA	Children (n = 325)			Adolescents (n = 265)		
	> 1SD y < 2SD *n* (%)	> 2SD *n* (%)	> CS *n* (%)	> 1SD y < 2SD *n* (%)	> 2SD *n* (%)	> *CS* *n* (%)
Anxiety	42 (12.9)	4 (1.2)	108 (33.2)	53 (20.0)	3 (1.1)	114 (43.0)
Depression	27 (8.3)	7 (2.2)	74 (22.8)	38 (14.3)	15 (5.7)	90 (34.0)
Rage control problems	68 (20.9)	15 (4.6)	140 (43.1)	53 (20.0)	9 (3.4)	95 (35.8)
Problems with emotional regulation	47 (14.5)	5 (1.5)	98 (30.2)	52 (19.6)	17 (6.4)	100 (37.7)
Rebellious behaviour	49 (15.1)	56 (17.2)	152 (46.8)	40 (15.1)	20 (7.5)	122 (46.0)
Somatic complaints	13 (4.0)	1 (0.3)	51 (15.7)	39 (14.7)	6 (2.3)	88 (33.2)
	> 1SD y < 2SD *n* (%)	> 2SD *n* (%)	< *CS* *n* (%)	> 1SD y < 2SD *n* (%)	> 2SD *n* (%)	< *CS* *n* (%)
Self-esteem^a^	24 (7.4)	5 (1.5)	99 (30.5)	32 (12.1)	7 (2.6)	105 (39.6)
Integration and social competence^a^	17 (5.2)	6 (1.8)	76 (23.4)	29 (10.9)	7 (2.6)	87 (32.8)
Awareness of the problems^a^	–	–	–	7 (2.6)	0 (0.0)	225 (84.9)

SD are the ones from the normal population of SENA technical manual.

*CS* The clinical sample score of the technical manual.

^a^The distance in standard deviations is below average.

Following, the results of the consequences of confinement are described first on children (8–12 years old) and then on the teenager sample (13–18 years old).

### Consequences of COVID confinement in children between 8 and 12 years old

First of all, Student t-tests are carried out as a sample in which the one of our study is compared with the technical standards of each of the SENA scales (Table 2).

**Table 2:** Differences between the study sample and SENA scales for children (between 8 and 12 years old) and adolescents (13–18 years old).

SENA	Children n = 325		*t*	*p*	*d*_*z*_	Adolescents n = 265		*t*	*p*	*d*_*z*_
	*M*	*DT*				*M*	*DT*			
Anxiety	2.40	0.82	2.49	**0.013**	0.14	2.81	0.89	5.64	**< 0.0001**	0.35
Depression	1.68	0.57	3.132	**0.002**	0.18	2.01	0.78	6.53	**< 0.0001**	0.40
Self-esteem	4.26	0.63	− 1.177	0.240	− 0.06	3.70	0.77	− 5.49	**< 0.0001**	− 0.34
Rage control problems	2.37	0.84	10.996	**< 0.0001**	0.61	2.42	0.84	7.75	**< 0.0001**	0.48
Problems with emotional regulation	2.25	0.89	4.794	**< 0.0001**	0.27	2.46	0.99	6.83	**< 0.0001**	0.42
Integration and social competence	3.99	0.63	− 2.725	**0.007**	− 0.16	3.81	0.68	− 7.25	**< 0.0001**	− 0.44
Rebellious behaviour	1.95	0.83	13.510	**< 0.0001**	0.75	1.99	0.83	6.19	**< 0.0001**	0.39
Somatic complaints	1.68	0.58	− 5.571	**< 0.0001**	− 0.31	2.01	0.80	2.20	**0.027**	0.03
Awareness of the problems						2.40	0.75	3.50	**0.001**	0.21

Statistically significant differences (*p* < .05) are highlighted in bold

As can be seen, once the confinement week is over, there are already significant differences concerning the scales in every dimension besides self-esteem. In all of them, apart from somatic complaints, the clinical situation of children between 8 and 12 years old is significantly worse than the sample of the scales. They show more rage control problems and rebellious behavior with medium effect size. They also show more anxiety, depression, problems with emotional regulation, somatic complaints and worse integration and social competence with low effect size.

The effect sizes found here are higher than the ones published in the technical manual of SENA^37^ regarding the differences between the scores of normal population and the clinical sample in rage control problems (d_z_ = 0.61 vs d_clinical_ = 0.39) and rebellious behaviour (d_z_ = 0.75 vs d_clinical_ = 0.35).

### Consequences of COVID confinement in teenagers from 13 to 18 years old

As can be seen in Table 2, after being confined for a week, teenagers show significant differences regarding the scales in all nine evaluated scales. In all of them, besides awareness of the problems, the clinical condition of teenagers between 13 and 18 years old is significantly worse. All effect sizes are low.

The effect sizes of the present study are greater than the ones published in the technical manual of SENA^37^ regarding the differences between the scores of the normal sample and the clinical one in Anxiety (d_z_ = 0.35 vs d_clinical_ = 0.26), Depression (d_z_ = 0.40 vs d_clinical_ = 0.34), Self-esteem (d_z_ = -0.34 vs d_clinical_ = 0.22), Rage Control Problems (d_z_ = 0.48 vs d_clinical_ = 0.39), Problems with Emotional Regulation (d_z_ = 0.42 vs d_clinical_ = 0.39), Integration and Social Competence (d_z_ = − 0.44 vs d_clinical_ = 0.40), Rebellious Behaviour (d_z_ = 0.39 vs d_clinical_ = 0.26).

### Differences between children and teenagers in Covid-19 confinement

In relation to the second goal, in order to compare data on children and teenagers, the scores of all the evaluated dimensions were standardised taking the average and standard deviation of the SENA scales for children (8–12 years old) and teenagers (13–18 years old). Then, a *MANOVA* was carried out with all the mental health variables set out in the study and shared with both groups, that is, all of them except for awareness of the problems.

The differences turned out to be significant (*Pillai’s* *trace* = 0.157, *F* = 13.551, *p* = 0.0001, *η*^2^ = 0.157) with a high effect size. The inter subject tests can be seen in Table 3. Teenagers show higher levels of anxiety, depression, problems with emotional regulation and somatic complaints, as well as lower self-esteem and integration and social competence levels than children. As for children, their sample reaches higher scores in rebellious behaviour than teenagers’. All effect sizes are low, except in Rebellious Behaviour where the effect size is medium.

**Table 3:** Differences in the state of mental health between children and adolescents during confinement.

SENA	Children (n = 325)		Adolescents (n = 265)		*F*	*p*	*η*_*p*_^2^
	*M*	*SD*	*M*	*SD*			
Anxiety	0.100	0.72	0.273	0.79	7.810	**0.005**	0.013
Depression	0.116	0.67	0.347	0.87	13.416	**< 0.0001**	0.022
Self-esteem	− 0.044	0.67	− 0.261	0.78	13.409	**< 0.0001**	0.022
Rage control problems	0.492	0.81	0.373	0.78	3.268	0.071	0.006
Problems with emotional regulation	0.206	0.77	0.382	0.91	6.440	**0.011**	0.011
Integration and social competence	− 0.091	0.61	− 0.324	0.73	17.916	**< 0.0001**	0.030
Rebellious behaviour	0.908	1.21	0.358	0.94	36.664	**< 0.0001**	0.059
Somatic complaints	− 0.173	0.56	0.108	0.79	25.413	**< 0.0001**	0.041

Statistically significant differences (*p* < .05) are highlighted in bold

### Differences by gender and level of education in children between 8 and 12 years old

Regarding the third goal, ANOVAs of a factor with SENA dimensions based on gender and educational cycle were carried out (Table 4).

**Table 4:** Differences by gender and educational level in the mental health of children between 8 and 12 years old during confinement.

SENA	Female (n = 168)		Male (n = 156)		*F*	*p*	*η*_*p*_^*2*^	Middle level (n = 141)		Upper level (n = 173)		*F*	*p*	*η*_*p*_^*2*^
	*M*	*SD*	*M*	*SD*				*M*	*SD*	*M*	*SD*			
Anxiety	2.45	0.85	2.35	0.79	1.119	0.291	0.003	2.40	0.86	2.40	0.79	0.000	0.992	0.000
Depression	1.72	0.63	1.63	0.51	1.879	0.171	0.006	1.73	0.62	1.64	0.53	1.749	0.187	0.006
Self-esteem	4.23	0.63	4.29	0.63	0.706	0.402	0.002	4.26	0.64	4.27	0.62	0.025	0.875	0.000
Rage control problems	2.32	0.83	2.42	0.85	1.204	0.273	0.004	2.48	0.88	2.30	0.80	3.577	0.059	0.011
Problems with emotional regulation	2.21	0.88	2.29	0.91	0.640	0.424	0.002	2.42	0.96	2.12	0.81	9.061	**0.003**	0.028
Integration and social competence	4.04	0.64	3.95	0.61	1.649	0.200	0.005	3.95	0.65	4.05	0.61	1.778	0.183	0.006
Rebellious behaviour	2.00	0.88	1.90	0.76	1.107	0.294	0.003	1.96	0.78	1.95	0.84	0.019	0.889	0.000
Somatic complaints	1.74	0.65	1.62	0.50	3.461	0.064	0.011	1.70	0.60	1.65	0.56	0.621	0.431	0.002

Statistically significant differences (*p* < .05) are highlighted in bold.

The middle level comprises 3rd and 4th elementary (8–10 years old). The upper level 5th and 6th elementary (10–12 years).

There are not significant differences regarding gender in any of the nine evaluated variables in children who are in quarantine. According to the educational level, the youngest of the middle elementary level revealed greater problems of emotional adjustment than those of the upper level (*F* = 9.061, *p* = 0.003, *η*^2^ = 0.028) with a low effect size.

### Differences by gender and level of education in teenagers between 13 and 18 years old

Regarding gender, the results show that there are significant differences between teenagers (Table 5), with females presenting higher levels of anxiety, less self-esteem, more problems with emotional regulation and more somatic complaints than their masculine counterparts. Then again, males show a significantly lower score on integration and social competence than female**s**. All effect sizes are low.

**Table 5:** Differences by gender and education in the mental health of teenagers between 13 and 18 years old during confinement.

SENA	Female (n = 168)		Male (n = 96)		*F*	*p*	*η*_*p*_^2^	ESO (n = 153)		Baccalaureate (n = 89)		Vocational training (n = 23)		*F*	*p*	*η*_*p*_^2^
	*M*	*SD*	*M*	*SD*				*M*	*SD*	*M*	*SD*	*M*	*SD*			
Anxiety	2.96	0.81	2.53	0.97	14.865	**< 0.0001**	0.054	2.58	0.90	3.09	0.77	3.22	0.86	12.674	**0.0001**	0.088
Depression	2.08	0.77	1.89	0.79	3.560	0.060	0.013	1.85	0.74	2.21	0.80	2.33	0.73	8.524	**0.0001**	0.061
Self-esteem	3.61	0.79	3.88	0.71	7.696	**0.006**	0.029	3.86	0.73	3.46	0.80	3.53	0.63	8.676	**0.0001**	0.062
Rage control problems	2.42	0.84	2.40	0.84	0.061	0.805	0.000	2.44	0.84	2.37	0.79	2.50	1.04	0.281	0.756	0.002
Problems with emotional regulation	2.56	0.98	2.27	1.00	5.208	**0.023**	0.019	2.32	1.00	2.58	0.91	2.88	1.08	4.307	**0.014**	0.032
Integration and social competence	3.88	0.66	3.69	0.71	4.658	**0.032**	0.017	3.84	0.65	3.77	0.72	3.70	0.75	0.629	0.534	0.005
Rebellious behaviour	1.94	0.81	2.05	0.86	0.906	0.342	0.003	2.05	0.87	1.90	0.77	1.88	0.83	1.203	0.302	0.009
Somatic complaints	2.18	0.79	1.94	0.79	5.521	**0.020**	0.021	1.95	0.78	2.30	0.80	2.29	0.80	6.463	**0.002**	0.047
Awareness of the problems	2.96	0.81	2.53	0.97	14.865	**< 0.0001**	0.054	2.58	0.90	3.09	0.77	3.22	0.86	12.674	**0.0001**	0.088

Statistically significant differences (*p* < .05) are highlighted in bold.

*ESO* Secondary, Middle School, *Baccalaureate* Sixth Forma, High School.

Regarding the level of education, there are significant differences among anxiety, depression, self-esteem, and awareness of the problems with a medium effect size, and also in problems with emotional regulation, and somatic complaints with a low effect size. In all of them, teenagers who study in ESO show a better clinical condition that the ones who study in high school and professional training, except for the scale of awareness of the problems (Table 6).

**Table 6:** Significance of the post-hoc differences (DMS test) between pair averages depending on the teenagers’ academic course during confinement.

SENA	ESO vs baccalaureate	ESO vs vocational training	Baccalaureate vs vocational training
	*p*	*p*	*p*
Anxiety	**< 0.0001**	**0.001**	–
Depression	**< 0.0001**	**0.005**	–
Self-esteem	**< 0.0001**	**0.050**	–
Rage control problems	–	–	–
Problems with emotional regulation	–	**0.011**	–
Integration and social competence	–	–	–
Rebellious behaviour	–	–	–
Somatic complaints	**0.001**	–	–
Awareness of the problems	**0.016**	**0.026**	–

Only statistically significant differences are reported.

*ESO* Secondary, Middle School.

## Conclusions

The results of the present report show that the strict confinement situation of children and teenagers already reveal, from 8 to 10 days, significant consequences on the mental health of both of them, although we still do not know the long-term effect (Hypothesis 1). It appears that the consequences of confinement on children are mostly in the affective area, this also being reflected at the behavioural level. They show problems of rebellious behaviour (*d*_*z*_ = 0.75), rage control (*d*_*z*_ = 0.61) and emotional regulation (*d*_*z*_ = 0.27) to a greater extent. As opposed to adults, children do not clearly identify these altered conditions in themselves, and it is frequent that symptoms like irritability or aggression appear as a warning signal of more chronic disorders for this age group. We have also discovered that during confinement they showed higher levels of anxiety (*d*_*z*_ = 0.14), depression (*d*_*z*_ = 18), and less integration and social competence (*d*_*z*_ = 16), although with lower effect sizes. However, it should be pointed out that such high percentages as 33.2% and 22.8% of the children in confinement score higher than the clinical sample of SENA on anxiety and depression respectively.

The only variable that revealed improvement in children during confinement was somatic complaints (*t* = − 5.571, *gl* = 324, *p* = 0.0001, *d*_*z*_ = − 0.31). One possible explanation is that somatic complaints usually appear as an involuntary expression of psychological discomfort that children use to seek attention or affection from their parents. Franco, Pérez and de Dios^47^ discover differences between parenting styles and the somatization of their children, proving that parents with less disciplined educational behaviour or situations in which one of the parents takes fewer caring tasks, increase the somatization in children. In our results, somatic complaints in children decreases, perhaps because parents are closer and spend more time with their children.

In the case of teenagers, there are significant differences regarding the scales of every evaluated variable (anxiety, depression, rebellious behaviour, somatic complaint problems with emotional regulation and rage control) being the effect size average except in awareness of the problems and somatic complaints, which is low.

In all the dimensions, the results show a worse clinical situation than in teenagers after being between 8 and 10 days in confinement, except in awareness of the problems (*t* = 3.50, *gl* = 264, *p* = 0.001, *d*_*z*_ = 0.21), that is, they were more aware during confinement that some things were not going well, that they were having a difficult time, so they could have needed help (Hypothesis 2).

Regarding gender, the results point in the same direction as the ones found by Zhou et al.^3^ in Chinese teenagers: Spanish girls between 13 and 18 years old have been more affected by confinement than boys (Hypothesis 3). They show more anxiety, less self-esteem, more problems with emotional regulation and more somatic complaints. As for male teenagers, they show lower levels of integration and social competence. Because of this, it seems they have greater difficulty being accepted and loved by others than females through the methods which social interaction in confinement allow (video calls, chats, phone calls, etc.). This might well be because teenage girls tend to use on-line means of socialization more, give more importance to them than boys do, and they affect their well-being more^48^. Consequently, they are more used to this socializing agent than boys, who use digital media more often for gaming^48^. Nevertheless, confinement does not seem to have a differential impact based on gender in children between 8 and 12 years old.

Regarding the school year, Zhou et al.^3^ discovered that teenagers during years with more academic pressure in students showed more anxiety and depression, since the COVID-19 outbreak interrupted their normal learning process. In our study the results are similar. Teenagers in high school (prior to examinations for University admission) showed lower levels of self-esteem and more anxiety, depression and somatic complaints than middle-school students. Professional training students also showed worse symptomatology (more anxiety, depression, problems with emotional regulation and less self-esteem) than the ones in middle school. Professional training students noticed how their internship came to a halt due to coronavirus, perhaps the part of their studies which they consider the most important and the one they value the most in this kind of education. Without a doubt, this meant a period of uncertainty to them since they did not know the terms and conditions in which they could carry out their studies. In short, the differences in teenagers’ mental health took into account high school and professional training students, as well as those in middle school, a school age that does not imply great academic pressure in Spain (Hypothesis 4).

In children between 8 and 12 years old, there were only differences in problems with emotional regulation, where middle-school students reached a higher score than the ones in high school. This coincides with the study that indicates that emotional control grows with age and that not until they are 10 years old, are children mature enough to understand states such as emotional ambivalence (experimenting contradictory emotions in the same situation)^49,50^. This would doubtlessly make emotional understanding and the ability to adequately face the confinement situation difficult for young children.

The present report represents a first approach to the consequences of confinement on Spanish children and teenagers, which, nevertheless, must be interpreted in the light of some limitations. First of all, the selection of the sample was for the sake of convenience and following snowball sampling. However, in future confinement situations, a probability sampling should be obtained, since the sample of the present study is not representative of Spanish children and adolescents, and therefore, special caution should be exercised in the interpretation of the results. Besides, the evaluation was transversal, which prevents establishing causal relationships. It should also be noted that the results displayed here only comprise the consequences of the first 8–10 days of confinement. This is the research, as far as we know, with the earliest evaluation in the confinement of minors. And from what we have found here, it seems that its effects are felt from the first week. Its long-term evolution still needs to be established. In this sense, the detection and intervention of children and adolescents at risk, when identified early, can help prevent the onset or worsening of psychopathologies^28^.

In short, looking at these results, it seems clear that strict confinement situations affect the mental health of children and teenagers between 8 and 18 years old. And it seems that the impact of confinement is different in children and adolescents, at least in the early stages of confinement. And, as far as we know, all previous research evaluating both age groups in Europe has done so jointly. Future research should study the mental health of minors according to their stage of development, as this could influence a different process of return to normality, different needs and vulnerabilities and/or the design of specific support programs.

Our results suggest a very early impact of strict home confinement on children and adolescents, which has implications for health policies. Countries which are less restrictive in their COVID-19 containment measures, such as Germany, appear to have received less impact on the mental health of minors^28^. In addition, the literature on the topic reflects that the COVID-19 pandemic affects young people with and without any underlying physical or mental health conditions, and that they experience feelings and emotions similar to those experienced by adults^16^. This indicates the need to carefully balance strict confinement measures with measures needed to curb the spread of contagious diseases. Our results—along the same lines as other recent studies in Germany, China or the USA^24,28,51^—make the need for specific mental health interventions for children and adolescents explicit. Future research should study prevention strategies for the deterioration of mental health and support in the promotion of mental health from the beginning of periods of confinement to cushion its impact.

## Data Availability

The data presented in the study are available from https://www.doi.org/10.17605/OSF.IO/RZ8AU.
